# Impact of Extreme Heat on Emergency Department Admissions for Childhood and Adult Asthma: An Evaluation of Earth Observations and Heat Wave Definitions

**DOI:** 10.1029/2025GH001501

**Published:** 2026-05-06

**Authors:** B. Corpuz, E. Scott, B. F. Zaitchik, S. Zeger, D. Waugh, A. Balasubramanian, J. Madrigano, K. Koehler, R. Koehl, M. McCormack

**Affiliations:** ^1^ Department of Earth and Planetary Sciences Johns Hopkins University Baltimore MD USA; ^2^ Department of Biostatistics Johns Hopkins University Bloomberg School of Public Health Baltimore MD USA; ^3^ Division of Pulmonary and Critical Care Medicine Johns Hopkins University School of Medicine Baltimore MD USA; ^4^ Department of Environmental Health and Engineering Johns Hopkins University Bloomberg School of Public Health Baltimore MD USA

## Abstract

Extreme heat has been associated with adverse health outcomes, yet its impact on asthma exacerbations remains understudied. This is, in part, due to data limitations: research that relies on weather station records and aggregated health statistics cannot resolve fine‐scale differences in heat impacts. This study investigates the association between heat wave definitions and summertime asthma‐related emergency department visits in Baltimore, Maryland from 2016 to 2022, including 819 adult and 695 pediatric exacerbations. Using geocoded electronic health records and air temperature measurements at several spatial resolutions, we applied a case‐crossover design with conditional logistic regressions at the census block group and tract levels. We found strong associations between asthma exacerbations and nighttime heat wave definitions based on relative thresholds of minimum temperatures when census block group or tract level temperature estimates were used. These relationships were significant for both age groups and showed elevated risks in socially vulnerable areas. In contrast, heat wave definitions derived from the city's primary National Weather Service synoptic weather station show associations between asthma and daytime heat extremes, suggesting that the character of the heat hazard depends on the scale at which it is defined. The extreme heat event definition used by Baltimore City's Code Red system showed no significant association with exacerbations. These findings highlight the importance of data resolution in shaping health inferences related to extreme heat in urban environments. Further, this study demonstrates that, regardless of spatial scale, extreme heat is associated with asthma exacerbations in both age groups.

## Plain Language Summary

Extreme heat is known to impact human health, but its effects on asthma are understudied. This study investigated the relationship between heat wave definitions and asthma‐related emergency department visits in Baltimore from 2016 to 2022, including 819 adult and 695 pediatric cases. By combining geocoded medical records with air temperature measurements at several spatial resolutions, we found that heat wave definitions based on elevated minimum nighttime temperatures were associated with asthma exacerbations in both age groups especially in socially vulnerable neighborhoods. These associations were observed when using high resolution temperature data, which capture neighborhood‐level heat exposure. In cities like Baltimore, the urban heat island effect is more pronounced at night, resulting in sustained elevated nighttime temperatures. This highlights the importance of high‐resolution temperature data in detecting localized heat exposure. In contrast, heat wave definitions from coarse resolution synoptic weather stations captured daytime heat impacts, but missed nighttime effects. Our findings emphasize the value of granular health and temperature data in identifying localized heat impacts on asthma. Baltimore's heat alert systems rely on daytime heat indices, which may underestimate the full extent of heat exposure. Incorporating nighttime metrics into public health warnings could strengthen heat preparedness and better protect vulnerable populations.

## Key Points

- Extreme heat events are associated with increases in childhood and adult asthma‐related emergency department visits in Baltimore
- In locally‐resolved heat analysis, heat wave metrics based on nighttime temperature best capture asthma risk
- The impact of extreme heat on asthma‐related emergency department visits is greater in socially vulnerable neighborhoods

## 1 Introduction

Recent decades have seen impressive progress in the diversity and distribution of environmental sensing technologies, including high resolution airborne and satellite‐borne imaging, personal monitoring devices, and micro‐networks of low‐cost sensors. These advances offer the potential for tremendous societal benefit across scales, including characterizing risks to human health (Cracknell, 2018; Dubovik et al., 2021; Gong et al., 2013). High resolution environmental monitoring, when paired with health data, allows for robust analysis of associations between environmental conditions and health outcomes. However, as the resolution of environmental data has improved, the limitations of available health data have become a greater and greater constraint on studies that apply Earth Observations (EO) for health. This is particularly true for community‐scale health studies, where active health surveillance can be limited, and privacy concerns often require aggregation of health data to coarser spatial scales. While such data are valuable for many health applications, their resolution can limit their utility for analyses aiming to capture neighborhood‐level variability (Bilheimer & Klein, 2010; Keshta & Odeh, 2021). This becomes especially challenging in urban settings, where spatial heterogeneity in climate and environmental conditions can influence health outcomes.

These limitations in health data present a barrier to EO applications to environmental justice that cuts in two ways: newer high resolution EO are not being used to their full power, and the value of taking observations to higher resolution cannot be fully characterized in the absence of spatially resolved health data. In this study, we overcome this challenge by using geocoded electronic health records from the Johns Hopkins Precision Medicine Analytics Platform (PMAP), which includes medical encounter data for patients served by the hospitals within Johns Hopkins Medicine. This allows us to merge health records with EO at scales as fine as the patient's street address, enabling detailed analyses of local conditions and health outcomes. We use this harmonized data set to evaluate the impact of extreme heat on asthma exacerbations.

Asthma, a chronic respiratory condition characterized by shortness of breath, wheezing, and chest tightness, is the most common lung disease found in children and affects people of all ages (Han et al., 2023). In the United States alone, 7.7% of the population, which corresponds to almost 25 million individuals, are diagnosed with asthma (CDC, 2023). Previous studies have suggested associations between extreme heat and an increased risk of asthma hospital admissions and hospitalization (Deng et al., 2023; Han et al., 2023; Makrufardi et al., 2023; Soneja et al., 2016). However, these studies rely on temperature data from the nearest weather station, which provide a single time series of weather data that fails to capture spatial variability within urban areas (Deng et al., 2023; Soneja et al., 2016). While National Weather Service (NWS) synoptic weather stations are a widely used source for temperature data, their coarse spatial resolution limits their ability to capture fine‐scale temperature variations necessary for detailed, neighborhood‐level analyses (Fang et al., 2021; Schinasi et al., 2022; Soneja et al., 2016). To improve upon this, our study incorporates high‐resolution air temperature measurements alongside NWS data, enabling more precise spatial analyses and comparisons.

Further, the health data used in urban asthma studies are rarely disaggregated to fine spatial scales, such as the census block group, limiting the precision of analyses. Baltimore City has one of the highest asthma prevalence rates in the country, disproportionately affecting low‐income and minority communities, and reports the highest rate of emergency department (ED) visits in the state of Maryland (Baltimore City Health Department, 2017; Pollack et al., 2023). These disparities underscore the need for high‐resolution analyses to better understand how variations in local heat exposure contribute to asthma exacerbations. By integrating geocoded medical records with high‐resolution daily, maximum and minimum air temperature measurements, we conducted a highly spatially resolved analysis of nighttime and daytime extreme heat impacts on asthma exacerbations across the city of Baltimore.

There is reason to expect that resolving local heat conditions within Baltimore is relevant when assessing asthma risk. Like many cities, Baltimore exhibits an urban heat island (UHI) in which inner city temperatures can be several degrees higher than those in surrounding areas, particularly at night (Corpuz et al., 2024; Shi et al., 2021). Baltimore is also a deeply segregated city in economic and racial terms, with high social vulnerability found in inner city neighborhoods, many of which experience elevated temperatures and elevated rates of asthma (Pollack et al., 2023). Studies relying on aggregated climate or health data sets risk overlooking these fine‐scale spatial patterns of vulnerability, exposure, and health outcomes, and may result in an incomplete picture of the relationship between heat hazards and asthma burden.

Another challenge for studies examining the health impacts of heat extremes is how one defines extreme heat. Heat waves are generally defined as prolonged periods of unusually high temperatures, though the exact definition can vary depending on the durations and thresholds used. A common approach is to treat daily ambient temperature (or some derived heat metric) as a continuous variable and diagnose relationships between daily heat values and health outcome in time‐synchronous or time‐lagged analysis (Rothfusz, 1990; Steadman, 1979). Other studies have modeled temperature as a continuous exposure using distributed lag non‐linear models, which account for cumulative effects of environmental exposures such as heat (Gasparrini et al., 2015; Schinasi et al., 2022). In the present study, we applied a heatwave‐based framing for extreme heat, treating extreme heat as a discrete event defined by thresholds in temperature intensity and duration. This aligns with prior research examining associations between heatwave events and health outcomes (Anderson & Bell, 2011; Frich et al., 2002; Hansen et al., 2008; Lyon, 2009; Rey et al., 2007; Robinson, 2001; Russo et al., 2017). Using a heatwave approach introduces the question of how one can most meaningfully define a heatwave for a specific health outcome of interest. Defining heatwaves remains complex, requiring decisions on duration, temperature thresholds, whether to focus on daytime, nighttime, or average temperatures, and whether extreme heat should be defined in absolute (e.g., >38°C) or relative terms (e.g., >95% of historic records at that location).

To address this complexity, we tested 11 heatwave definitions drawn from recent literature. These definitions included those that rely on relative thresholds, where extreme temperatures are determined based on percentile‐based deviations from the regional historical norms (Anderson & Bell, 2011; Lyon, 2009; Rey et al., 2007), as well as those that use absolute thresholds, defining heat waves based on fixed temperatures values that are considered dangerous to human health (Frich et al., 2002; Hansen et al., 2008; Robinson, 2001; Rothfusz, 1990; Russo et al., 2017; Steadman, 1979). Analyzing different definitions allows us to provide a better understanding of asthma risks associated with nighttime and daytime heat exposure, and to explore how different thresholds for heat intensity, duration, and cumulative impacts of prolonged heat exposure affect estimates of health risk. Although epidemiologic studies largely rely on daytime or maximum temperature metrics to assess heat‐related asthma risk, minimum temperatures are rarely assessed, resulting in limited understanding of how nighttime heat exposure contributes to asthma risk (Deng et al., 2023; Konstantinoudis et al., 2023; Yu et al., 2024). For each definition, we calculated a spatially distributed version of the heatwave index using high‐resolution temperature maps derived from a combination of in situ low‐cost temperature sensors and satellite data (Corpuz et al., 2024), and compared it to a spatially uniform estimate calculated using data from a NWS synoptic weather station.

To our knowledge, this is the first study to examine the impact of heat waves on asthma exacerbations using geocoded health records and varying spatial resolutions of air temperature data. In addition to providing meaningful target scales for EO‐health analysis in an environmental justice application, this study also helps identify appropriate heat wave definitions for urban health studies.

## 2 Methods

### 2.1 Study Area

This study focuses on Baltimore, Maryland, a mid‐sized metropolitan city in the eastern United States. As the largest city in the state of Maryland, Baltimore has a population of 585,708 (U.S. Census Bureau, 2020). The city spans geographic coordinates between 39.37° and 39.20° latitude and −76.71° and −76.53° longitude. Baltimore's urban setting and humid subtropical climate make it an ideal location to investigate the localized effects of extreme heat on public health.

### 2.2 Health Data

**Figure 1:**
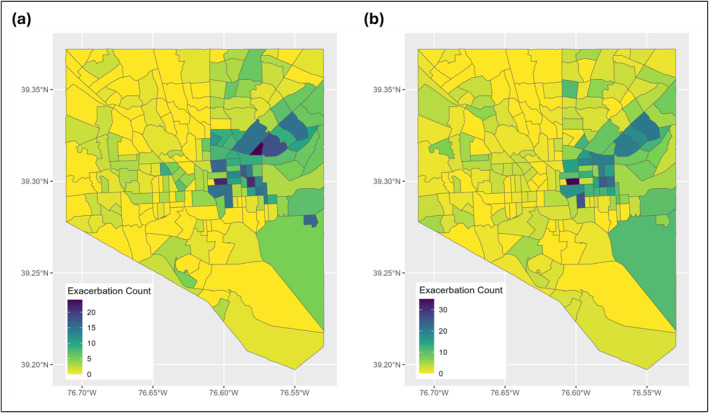
Spatial distribution of asthma exacerbation counts by census tract in (a) pediatric and (b) adult patients from 2016 to 2022. Note that Johns Hopkins Hospital is located in East Baltimore, which partly explains the spatial distribution of counts.

Daily, patient‐level ED visit data were obtained from Johns Hopkins hospitals in Baltimore through the Johns Hopkins PMAP. Severe asthma exacerbations were identified using ICD‐10 codes and included ED visits that resulted in inpatient care, hospital observation, or treatment in the ED. Since this case definition relies on ED visits requiring treatment or observation, asthma exacerbations evaluated in the ED that did not require hospitalization or extended observation are not included. This approach focuses the analysis on clinically significant events with documented medical evaluation, while acknowledging that milder episodes may not be represented. All cases were geocoded to the patient address level and subsequently aggregated to their respective census block group and census tracts for spatial analysis. Cases were categorized into two age groups: pediatric patients (<18 years old) and adult patients (≥18 years old), comprising 695 pediatric exacerbations and 819 adult exacerbations (Figure 1). To examine the impact of extreme heat on asthma exacerbations, this study analyzed data collected during the summer months of June, July, and August from 2016 to 2022.

### 2.3 Heat Data

**Figure 2:**
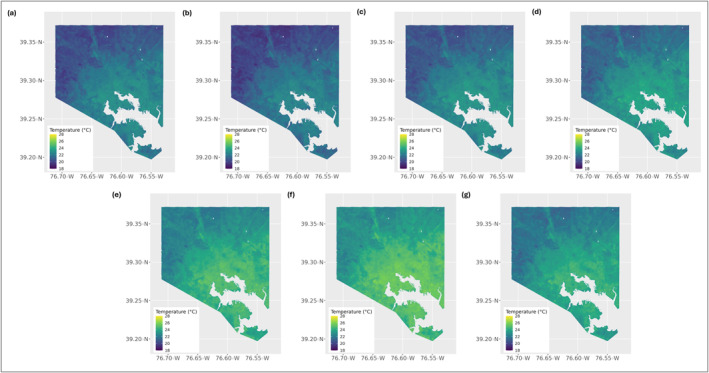
Daily minimum air temperatures averaged over the summer months for each year of the study period, derived from our high‐resolution air temperature data: (a) 2016, (b) 2017, (c) 2018, (d) 2019, (e) 2020, (f) 2021, and (g) 2022.

In a previous study, high‐resolution, 1‐m air temperature maps were developed for Baltimore using generalized additive models (GAMs) to predict daily maximum and minimum air temperatures for the summer months of 2016 on the basis of local landscape characteristics (Corpuz et al., 2024). Building on this method, the GAM framework was applied to generate daily, minimum (nighttime) and maximum (daytime) air temperature estimates for June, July, and August from 2017 to 2022 (Figure 2). The GAMs used to generate the daily minimum and maximum air temperatures achieved RMSE values of 0.75 and 0.72 and adjusted *R* ^2^ values of 0.84 and 0.81, respectively, based on model evaluation procedures reported in Corpuz et al., 2024. These high‐resolution air temperature measurements were subsequently aggregated to the census block group and tract levels using a spatially weighted average. To provide a comparison to standard weather station approaches, temperature data from the nearest NWS synoptic weather station located within the Thurgood Marshall Baltimore‐Washington International airport grounds were collected and assigned uniformly to all census block groups and tracts for each day in our study period (NOAA, 2024a).

In this study, we test 11 different heat wave indices (Table 1). These heat wave indices span the major categories used in heat‐health research, including absolute thresholds, percentile‐based thresholds, and duration‐based metrics, allowing us to capture different dimensions of heat exposure relevant to asthma (Kent et al., 2014; Smith et al., 2013). The relative and absolute thresholds used in this study follow established definitions often utilized in heat‐health literature which reflect conditions of high thermal stress for local populations. For example, absolute thresholds such as 35°C are commonly used in heat‐health studies because they approximate conditions at which heat index values exceed 40°C in humid subtropical climates like Baltimore's (Hansen et al., 2008; Russo et al., 2017). These definitions also differ in their clinical relevance; for example, duration‐based metrics such as HI07 capture prolonged heat exposure that may impose cumulative physiological stress, whereas percentile‐based metrics reflect anomalously hot days for which individuals may be less acclimated. Maximum‐temperature indices include HI01, HI03, HI05, HI07, HI08, and HI09; minimum‐temperature indices include HI02, HI04, and HI06; and mixed indices based on mean or composite temperatures include HI10 and HI11. Ten are previously published indices, including a daily‐maximum heat index method used by the Baltimore City government to issue Code Red Extreme Heat Alerts (HI11 in our Table 1). This heat index (HI11) uses the NWS Heat Index, which combines maximum air temperature and relative humidity using the Rothfusz heat index equation. Because it is derived from the NWS synoptic station at BWI airport, it reflects conditions at a single location rather than spatially varying heat exposures within the city (Rothfusz, 1990). Code Red Alerts trigger citywide public health responses, such as opening of cooling centers and providing of safety tips and heat‐related resources offered through 311 operators. We also added an index to provide a low threshold minimum temperature index (HI02 in Table 1).

**Table 1:** Summary of Heat Wave Definitions and Their Corresponding Criteria, Including Temperature Metrics, Thresholds, Durations, and Heat Wave Types

Heat wave indices (HI)	Temperature metric	Threshold	Duration	HI type	Reference
HI01	Maximum daily temperature	≥90th percentile	2+ consecutive days	Relative	Lyon (2009)
HI02	Minimum daily temperature	≥90th percentile	2+ consecutive days	Relative	*This study*
HI03	Maximum daily temperature	≥95th percentile	2+ consecutive days	Relative	Anderson and Bell (2011)
HI04	Minimum daily temperature	≥95th percentile	2+ consecutive days	Relative	Anderson and Bell (2011)
HI05	Maximum daily temperature	≥95th percentile	3+ consecutive days	Relative	Rey et al. (2007)
HI06	Minimum daily temperature	≥95th percentile	3+ consecutive days	Relative	Rey et al. (2007)
HI07	Maximum daily temperature	≥ average maximum daily temperature by 5°C of CNP	5+ consecutive days	Absolute	Frich et al. (2002)
HI08	Maximum daily temperature	≥35°C	2+ consecutive days	Absolute	Russo et al. (2017)
HI09	Maximum daily temperature	≥35°C	3+ consecutive days	Absolute	Hansen et al. (2008)
HI10	Maximum daily heat index (DHI); Minimum daily temperature	Max DHI ≥ 40.6°C and Min temp ≥ 26.7°C	2+ consecutive days	Absolute	Robinson (2001)
HI11	Maximum daily heat index (DHI)	Max DHI ≥ 40.6°C	1 day	Absolute	Rothfusz (1990); Steadman (1979)

To establish baseline climatic conditions, we used the most recent 30‐year climate normal period (CNP) (1991–2020), as defined by the National Oceanic and Atmospheric Administration (NOAA, 2024b), using NWS station data from the Thurgood Marshall Baltimore/Washington International (BWI) airport to calculate the statistical distribution of temperatures. As shown in Table 1, we emphasized heat wave definitions that incorporate maximum and minimum temperatures, fully utilizing the high‐resolution air temperature and NWS station‐derived measurements. To account for relative humidity in the daily heat index (DHI)‐based definitions HI10 and HI11, we used relative humidity measurements from the Parameter‐elevation Regressions on Independent Slopes Model (PRISM) Climate Group (PRISM Climate Group, 2024). The DHI‐based definitions HI10 and HI11 use the Rothfusz heat index equation to combine air temperature and relative humidity into a single measure of perceived heat; HI10 applies a joint threshold requiring both a maximum DHI and a minimum temperature, whereas HI11 relies solely on a maximum DHI threshold (Robinson, 2001; Rothfusz, 1990; Steadman, 1979). Note that the use of the NWS station to calculate 30‐year climate normal statistics means that the “relative” heatwave indices are calculated relative to the weather station percentiles rather than each census block or tract statistics.

### 2.4 Statistical Analysis

We applied a case‐crossover design by performing conditional logistic regressions to estimate odds ratios (ORs) for asthma exacerbations associated with heat wave exposure. Analyses were stratified by age group (pediatric and adult patients) and spatial resolution (census tract and block group levels). The primary exposure metrics included heat wave definitions derived from high‐resolution air temperature data and NWS station‐derived air temperature data.

To account for both the cumulative and delayed impacts of heat waves, we assessed whether each patient experienced a heat wave event within 7 days preceding the exacerbation event. Specifically, we estimated the association between extreme heat events occurring within the week preceding an asthma exacerbation and the odds of an asthma exacerbation. Heat wave events were determined using multiple definitions (Table 1), including both absolute and relative thresholds. These exposures were modeled as binary indicators, indicating the occurrence or non‐occurrence of a heat wave within the within the prior 7‐day period.

The case‐crossover design was selected for its suitability in studying the association between transient exposures and rare outcomes such as asthma exacerbations. Further, by allowing each patient to serve as their own control, this design effectively controls for time‐invariant individual factors that could confound the outcome and exposure in this study (Maclure, 1991). Control windows were selected to match the temporal pattern of the asthma exacerbation events, specifically focusing on the day of the week. We selected control windows that occurred one to 2 weeks prior to the start of the 7‐day period. Post‐event control windows were not used to avoid potential bias from treatment effects, as patients typically receive interventions such as medications or changes in management upon hospital presentation, and this treatment may alter the patient's inherent risk of subsequent exacerbations (Lu et al., 2008). Noting the potential for seasonal bias introduced by using only pre‐event control windows, we also considered symmetric bidirectional control windows and found the results to be similar. We report the pre‐event control results here, as centered windows may include post‐event days influenced by treatment.

Although it is possible that Air Quality Index (AQI) and relative humidity lie on the causal pathway between temperature and asthma exacerbations, it is also possible that each of these co‐exposures share a common meteorological cause (e.g., stagnation event, etc.). In consideration of these possibilities, and for consistency with prior literature, we ran models unadjusted for AQI and relative humidity, and subsequently, adjusted for AQI and relative humidity as a sensitivity analysis (Buckley et al., 2014; Deng et al., 2023; Reid et al., 2012; Yu et al., 2024). Air quality data were derived from the AirNow platform, which provided daily AQI values (AirNow, 2024). Relative humidity data were obtained from the PRISM Climate Group (PRISM Climate Group, 2024). To evaluate whether including relative humidity and AQI might introduce collinearity in the models, we examined correlations and variance inflation factors (VIFs). Correlations between heat‐wave events and both relative humidity and AQI were low, and VIFs for all predictors were well below commonly accepted thresholds, indicating no evidence of problematic multicollinearity. We also incorporated the Social Vulnerability Index (SVI) as an effect modifier for analyses conducted at the census tract level and Area Deprivation Index (ADI) for analyses conducted at the census block group level (Centers for Disease Control and Prevention, 2024; University of Wisconsin School of Medicine and Public Health, 2024). These indices were chosen because they capture distinct yet complementary dimensions of socioeconomic disparities across spatial units (Flanagan et al., 2011; Kind & Buckingham, 2018). The SVI, calculated at the census tract level, integrates factors such as socioeconomic status, household composition, minority status, and housing type (Flanagan et al., 2011). The ADI, which is available exclusively at the census block group level, focuses specifically on indicators like income, education, and employment (Kind & Buckingham, 2018). SVI, which is reported on a scale of 0–1, was scaled to represent a 0.1‐unit increase, while ADI which is measured on a 0–100 scale, was scaled to reflect changes per 10‐unit increase. The inclusion of both SVI and ADI ensures that our models account for spatially distinct patterns of socioeconomic vulnerability and deprivation, which are crucial for understanding and addressing disparities in environmental exposure and health outcomes. Because these indices capture different dimensions of vulnerability, their use as effect modifiers helps interpret whether social‐demographic factors captured by SVI or economic disadvantage captured by ADI more strongly influence heat‐related asthma risk at their respective spatial scales.

## 3 Results and Discussion

**Figure 3:**
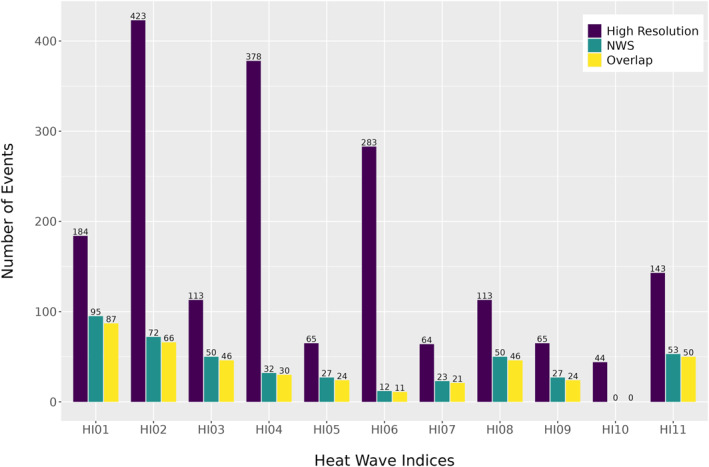
Heat wave events detected during summer months, June, July, and August, from 2016 to 2022 in Baltimore, by Heat Wave Indices (His) used in this study. Event counts are shown for each HI using high‐resolution, air temperature data (purple), synoptic NWS air temperature data (green), and events detected by both data sets (yellow).

A total of 695 pediatric exacerbations and 819 adult exacerbations occurred during the summer months of July and August in 2016 and from June to August in 2017–2022. Figure 3 illustrates the total number of heat wave events identified by each Heat Wave Index (HI) used in this study, derived using high resolution and synoptic NWS air temperature data. Across all HIs, high‐resolution temperature consistently detected more heat wave events than NWS data. For instance, between 44 and 423 heat wave events were identified using high resolution data, while the same HIs derived using NWS data identified between zero and 95 events. Because the high‐resolution and NWS air temperature data sets differ substantially in spatial resolution, comparing them allows us to evaluate how data‐source choice influences heat‐event classification, including the extent to which single‐station measures used in public health heat alerts may under‐represent spatially heterogeneous urban heat conditions. Indices based on relative thresholds, which define heat wave events as temperatures exceeding a percentile of historical conditions, generally detected more events compared to those based on absolute thresholds. Notably, HI10, which is based on an absolute daytime threshold, detected zero events when applied to NWS data, underscoring how coarser spatial resolution may fail to capture shorter or more localized episodes of extreme heat. The number of overlapping events, defined as those identified on the same dates by both data sources, also varied widely by indices, ranging from as few as 11–87 co‐occurring events. NWS undercount relative to high resolution temperature data was most dramatic for heat wave definitions based on nighttime values (HI02, HI04, and HI06), reflecting the fact that the urban heat island is greatest at night. This means that inner city neighborhoods are prone to cross nighttime temperature thresholds more frequently than suburban sites like the NWS location. Cross‐source comparisons and within‐source co‐occurrence matrices which further demonstrate the temporal agreement across HIs are provided in Figures S1 and S2 in Supporting Information S1. In addition, Figures S3 and S4 in Supporting Information S1 present co‐occurrence matrices of asthma exacerbation counts associated with HIs, illustrating that the number of asthma exacerbations corresponding to heat wave events varied across different definitions.

**Figure 4:**
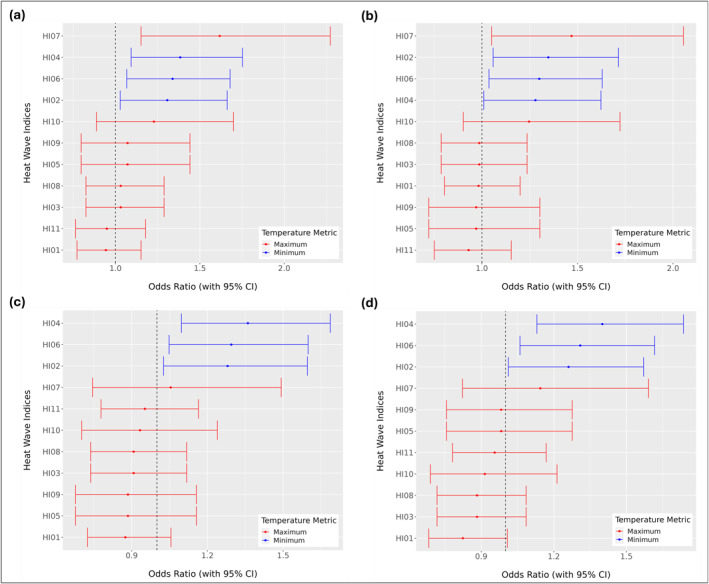
Odds ratios (ORs) for asthma exacerbations by heat wave definitions using high‐resolution, 1‐m air temperature data. Heat Wave Indices (HIs) derived from daily maximum temperature or heat index are shown in red, while HIs based on daily minimum temperature are shown in blue. Results are shown for pediatric (a, b) and adult (c, d) patients, at the census tract (a, c) and census block group (b, d) levels.

Our analyses using heat wave definitions derived from high‐resolution, 1‐m air temperature data revealed that nighttime heat wave definitions, particularly those based on relative thresholds of minimum temperatures were significantly associated with increased odds of asthma exacerbations (*α* < 0.05). This association was observed consistently among adult and pediatric patients across the census block group and census tract levels (Figure 4). These findings underscore the critical role of sustained nighttime heat exposure during the week preceding an asthma exacerbation in increasing the odds of experiencing an asthma event. Although this pattern is clear in Baltimore, it should be interpreted in the context of the city's humid subtropical climate and pronounced nocturnal UHI. Patterns may differ in arid or tropical climate where humidity, building design, and cooling practice vary. Even so, the key finding that prolonged nighttime heat elevates asthma risk is relevant when considering potential health burdens in urban environments experiencing increasing nighttime temperature.

Several mechanisms may help explain why warmer nights increase asthma symptoms. Higher temperatures can make the airways more sensitive by triggering nerves in the lungs that promote tightening of the bronchial muscles (Hayes et al., 2012). Heat has also been shown to increase airway irritation and inflammation, which can make breathing more difficult (Han et al., 2023). Indoors, warm nighttime conditions can encourage the growth of common allergens which are known triggers for asthma (Hashimoto et al., 2004). Warmer conditions can also coincide with higher levels of pollutants such as ozone, which have been shown in other studies to irritate the airways and amplify respiratory symptoms (Han et al., 2023). Together, these processes provide a biologically plausible explanation for why elevated nighttime temperatures may worsen asthma.

To help interpret the heat‐asthma associations across multiple metrics, it is important to note that HI01, HI03, HI05, HI07, HI08, and HI09 are daytime heat indices; HI02, HI04, and HI06 are nighttime hear indices; and HI10‐HI11 reflect mixed mean or composite temperatures. In pediatric patients at the census tract level, significant associations between heat wave exposure and asthma exacerbations were identified for HIs: HI07 (OR: 1.62; 95% CI: 1.15, 2.27), HI04 (OR: 1.38; 95% CI: 1.09, 1.75), HI06 (OR: 1.34; 95% CI: 1.07, 1.68), and HI02 (OR: 1.31; 95% CI: 1.03, 1.66) (Table 2). For adult patients at the census tract level, HI04 (OR: 1.36; 95% CI: 1.10, 1.69), HI06 (OR: 1.29; 95% CI: 1.05, 1.60), and HI02 (OR: 1.28; 95% CI: 1.03, 1.60) were significantly associated with increased odds of asthma exacerbations. Although the specific HI with the largest effect size varied slightly between age groups, the same set of nighttime based HIs were statistically significant across pediatric and adult patients, demonstrating consistent patterns of association. At the census block group level, the same set of heatwave definitions yielded significant associations. For pediatric patients, HI07 (OR: 1.47; 95% CI: 1.05, 2.05), HI04 (OR: 1.28; 95% CI: 1.01, 1.62), HI06 (OR: 1.30; 95% CI: 1.04, 1.63), and HI02 (OR: 1.35; 95% CI: 1.06, 1.71) were significantly associated with increased odds of asthma exacerbations. Among adults, significant associations with asthma exacerbations were found for HI04 (OR: 1.40; 95% CI: 1.13, 1.74), HI06 (OR: 1.31; 95% CI: 1.06, 1.62), and HI02 (OR: 1.26; 95% CI:1.01, 1.57).

**Table 2:** Odds Ratios (ORs) and 95% Confidence Intervals (CIs) for Asthma Exacerbations by Heat Index Definitions (HIs) and Resolution for Pediatric and Adult Patients in Baltimore, From 2016 to 2022

Heat wave indices (HI)	Spatial resolution	Age group	OR and 95% CI (High resolution)	OR and 95% CI (NWS)
HI01	Census Tract	Pediatric	0.94 (0.77, 1.15)	**1.32 (1.06, 1.63)**
HI01	Census Tract	Adult	0.87 (0.73, 1.06)	1.06 (0.87, 1.29)
HI01	Census Block Group	Pediatric	0.98 (0.80, 1.20)	**1.32 (1.06, 1.63)**
HI01	Census Block Group	Adult	0.82 (0.68, 1.01)	1.06 (0.87, 1.29)
HI02	Census Tract	Pediatric	**1.31 (1.03, 1.66)**	1.09 (0.87, 1.37)
HI02	Census Tract	Adult	**1.28 (1.03, 1.60)**	1.22 (0.98, 1.52)
HI02	Census Block Group	Pediatric	**1.35 (1.06, 1.71)**	1.09 (0.87, 1.37)
HI02	Census Block Group	Adult	**1.26 (1.01, 1.57)**	1.22 (0.98, 1.52)
HI03	Census Tract	Pediatric	1.03 (0.82, 1.29)	**1.56 (1.22, 1.99)**
HI03	Census Tract	Adult	0.91 (0.74, 1.12)	**1.28 (1.02, 1.60)**
HI03	Census Block Group	Pediatric	0.99 (0.79, 1.24)	**1.56 (1.22, 1.99)**
HI03	Census Block Group	Adult	0.88 (0.72, 1.09)	**1.28 (1.02, 1.60)**
HI04	Census Tract	Pediatric	**1.38 (1.09, 1.75)**	0.97 (0.76, 1.24)
HI04	Census Tract	Adult	**1.36 (1.10, 1.69)**	0.98 (0.78, 1.23)
HI04	Census Block Group	Pediatric	**1.28 (1.01, 1.62)**	0.97 (0.76, 1.24)
HI04	Census Block Group	Adult	**1.40 (1.13, 1.74)**	0.98 (0.78, 1.23)
HI05	Census Tract	Pediatric	1.07 (0.80, 1.44)	**1.50 (1.15, 1.95)**
HI05	Census Tract	Adult	0.89 (0.68, 1.16)	1.22 (0.95, 1.58)
HI05	Census Block Group	Pediatric	0.97 (0.72, 1.30)	**1.50 (1.15, 1.95)**
HI05	Census Block Group	Adult	0.98 (0.76, 1.28)	1.22 (0.95, 1.58)
HI06	Census Tract	Pediatric	**1.34 (1.07, 1.68)**	1.00 (0.70, 1.43)
HI06	Census Tract	Adult	**1.29 (1.05, 1.60)**	1.04 (0.77, 1.42)
HI06	Census Block Group	Pediatric	**1.30 (1.04, 1.63)**	1.00 (0.70, 1.43)
HI06	Census Block Group	Adult	**1.31 (1.06, 1.62)**	1.04 (0.77, 1.42)
HI07	Census Tract	Pediatric	**1.62 (1.15, 2.27)**	**1.74 (1.25, 2.42)**
HI07	Census Tract	Adult	1.05 (0.74, 1.49)	1.07 (0.77, 1.48)
HI07	Census Block Group	Pediatric	**1.47 (1.05, 2.05)**	**1.74 (1.25, 2.42)**
HI07	Census Block Group	Adult	1.14 (0.82, 1.59)	1.07 (0.77, 1.48)
HI08	Census Tract	Pediatric	1.03 (0.82, 1.29)	**1.56 (1.22, 1.99)**
HI08	Census Tract	Adult	0.91 (0.74, 1.12)	**1.28 (1.02, 1.60)**
HI08	Census Block Group	Pediatric	0.99 (0.79, 1.24)	**1.56 (1.22, 1.99)**
HI08	Census Block Group	Adult	0.88 (0.72, 1.09)	**1.28 (1.02, 1.60)**
HI09	Census Tract	Pediatric	1.07 (0.80, 1.44)	**1.50 (1.15, 1.95)**
HI09	Census Tract	Adult	0.89 (0.68, 1.16)	1.22 (0.95, 1.58)
HI09	Census Block Group	Pediatric	0.97 (0.72, 1.30)	**1.50 (1.15, 1.95)**
HI09	Census Block Group	Adult	0.98 (0.76, 1.28)	1.22 (0.95, 1.58)
HI10	Census Tract	Pediatric	1.23 (0.89, 1.70)	‐
HI10	Census Tract	Adult	0.93 (0.70, 1.24)	‐
HI10	Census Block Group	Pediatric	1.25 (0.90, 1.72)	‐
HI10	Census Block Group	Adult	0.91 (0.69, 1.21)	‐
HI11	Census Tract	Pediatric	0.95 (0.76, 1.18)	1.17 (0.89, 1.53)
HI11	Census Tract	Adult	0.95 (0.78, 1.16)	1.01 (0.81, 1.23)
HI11	Census Block Group	Pediatric	0.93 (0.75, 1.15)	1.17 (0.89, 1.53)
HI11	Census Block Group	Adult	0.96 (0.78, 1.17)	1.00 (0.81, 1.23)

*Note*. High resolution corresponds to HI definitions derived from 1‐m air temperature data, while NWS refers to National Weather Service (NWS) air temperature data from the BWI airport station. Estimates with *p*‐values <0.05 are marked as bold. A dash indicates insufficient data available. Rows corresponding to nighttime temperature‐based heat indices (HI02, HI04, HI06) are lightly shaded to aid visual interpretation.

Across the census block group and census tract levels, the same heat wave definitions were statistically significant for each age group. Every one of the significant heat wave definitions is based on minimum rather than maximum temperature, with the sole exception of HI07 for pediatric patients. We note that HI07 is a rarely occurring, extreme definition that indicates very prolonged (5+ days) heat conditions. Its statistical significance reflects the fact that it captures the largest heat events, and these events are typically also captured by other heat wave definitions.

For pediatric patients, narrower CIs were observed at the census block group level, indicating greater precision in those estimates. In adult patients, narrower CIs were found at the census tract level. However, within each age group, the differences in OR estimates between the census block group and census tract levels were small in magnitude, with all differences less than a 1.00, suggesting consistent results across spatial scales. These findings imply that urban heat studies can use the coarser scale without a substantial loss of precision.

The odds ratios for asthma exacerbations, adjusted for AQI are provided in Table S1 in Supporting Information S1. The addition of AQI and RH to the model did not result in a meaningful change to the unadjusted results in Table 2. Additionally, relative humidity and AQI were not statistically significant predictors of asthma exacerbations at either spatial scale or for either age group. This indicates that the observed associations between heat wave exposure and asthma exacerbations were robust to the addition of these co‐exposures to the model. Recent studies have also shown that associations between extreme temperature and asthma exacerbation persist even after adjusting for air pollution or aeroallergen exposures, suggesting that extreme temperature may be an independent predictor of asthma risk (Han et al., 2023). The lack of association may be partly due to the coarse spatial resolution of these data, which may not capture neighborhood‐level variations in air quality or humidity that are relevant to asthma risk.

**Figure 5:**
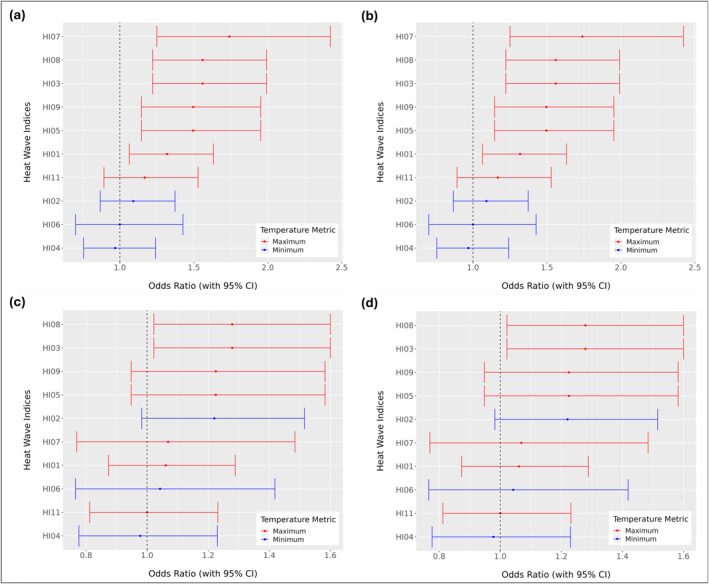
Odds ratios (ORs) for asthma exacerbations by heat wave definitions using synoptic NWS air temperature data. Heat Wave Indices (HIs) derived from daily maximum temperature or heat index are shown in red, while HIs based on daily minimum temperature are shown in blue. Results are shown for pediatric (a, b) and adult (c, d) patients, at the census tract (a, c) and census block group (b, d) levels.

Analyses using heat wave definitions based on NWS air temperature data from the BWI airport station found that daytime heat wave definitions were statistically significant (Figure 5). The following heat wave definitions were statistically significant for pediatric patients at the census tract and block group levels: HI07 (OR: 1.74; 95% CI: 1.25, 2.42), HI08 (OR: 1.56; 95% CI: 1.22, 1.99), HI03 (OR: 1.56; 95% CI: 1.22, 1.99), HI09 (OR: 1.50; 95% CI: 1.15, 1.95), HI05 (OR: 1.50; 95% CI: 1.15, 1.95), and HI01 (OR: 1.32; 95% CI: 1.06, 1.63). For adults, the statistically significant definitions were HI08 (OR: 1.28, 95% CI: 1.02, 1.60) and HI03 (OR: 1.28; 95% CI: 1.02, 1.60), emphasizing the prominence of daytime maximum temperatures in these analyses. Similar to the results from using high‐resolution air temperature data, the results for models that controlled for relative humidity and AQI are provided in Table S1 in Supporting Information S1. Similar to the results from using high‐resolution air temperature data, all models controlled for relative humidity and AQI. Neither variable was a statistically significant predictor of asthma exacerbations at either spatial scale or for either age group, confirming that the observed associations between heat wave exposure and asthma exacerbations were robust despite the addition of potential co‐exposures.

As we have seen, heat wave definitions based on NWS synoptic station air temperature data were primarily associated with daytime heat wave events, while high‐resolution, 1‐m air temperature data revealed statistically significant associations between asthma exacerbations and nighttime heat wave events. This distinction underscores the importance of spatial granularity in environmental health research. High‐resolution data captured the localized effects of sustained nighttime heat, consistent with evidence that elevated nighttime temperatures disrupt recovery from daytime heat exposure and disproportionately affect vulnerable populations (Basu & Samet, 2002). As shown in Figure 3, high‐resolution data consistently detected a greater number of heat waves across all HIs compared to NWS data. On the other hand, coarse resolution data emphasized maximum daytime temperatures, which, although indicative of broader heat wave conditions, may miss localized impacts such as urban heat islands or neighborhood‐specific vulnerabilities. Differences in heatwave detection between high‐resolution, 1‐m air temperature data and NWS synoptic station air temperature data were reflected in the number of asthma exacerbations captured during heatwave periods. Asthma exacerbations occurred on heatwave days across all definitions and data sets, with the highest number of cases observed under nighttime‐based HIs derived from high‐resolution air temperature data (Figure 3 in Supporting Information S1).

These findings highlight the complementary nature of both data sets while emphasizing the unique insights afforded by high‐resolution temperature data. Synoptic weather station data provide valuable insights into regional trends, while high‐resolution EO data reveal local effects essential for understanding city‐scale, neighborhood health risks. For city‐scale analyses, high‐resolution temperature data are particularly valuable as they allow for more precise analyses of heat‐health dynamics in urban environments and can support targeted interventions.

Baltimore City, the focus of our study, issues Code Red Extreme Heat Alerts based on a heat index definition derived from NWS thresholds which rely on a heat index threshold calculated using maximum daytime temperatures. Code Red Alerts trigger citywide public health responses, such as opening of cooling centers and providing of safety tips and heat‐related resources offered through 311 operators. However, this heat index definition, represented as HI11, was not statistically significant for predicting asthma exacerbations in either age group. H11 relies solely on daytime heat‐index values and does not capture nighttime heat exposure, which our results suggest is more strongly associated with asthma exacerbations; this likely accounts for the lack of association observed for H11. In contrast, all heat wave definitions based on minimum temperatures were statistically significant in the high‐resolution analysis, indicating that nighttime heat wave events are strongly associated with higher odds of asthma exacerbations. Nighttime heat metrics are especially critical for tailoring public health responses, as elevated nighttime temperatures hinder physiological recovery from daytime heat exposure and disproportionately affect vulnerable populations such as children and the elderly (Basu & Samet, 2002; Heaviside et al., 2017; Hondula et al., 2015). This supports our suggestion that alternative heat wave definitions, particularly those incorporating minimum nighttime temperatures, could enhance the effectiveness of public health responses related to extreme heat. Expanding the criteria for Code Red alerts to include nighttime measures and extending cooling center hours further into the evening could improve Baltimore City's capacity to mitigate heat‐related health risks.

**Figure 6:**
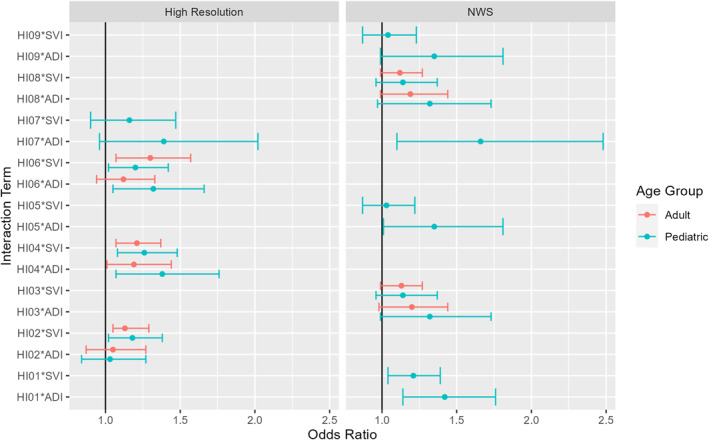
Odds ratios (ORs) and 95% confidence intervals (CIs) for interaction effects between Heat Wave Indices (HIs) and social vulnerability (SVI) or area deprivation indices (ADI), stratified by data source, spatial resolution and age group. The figure displays all combinations of heatwave definition, spatial resolution, and age group with significant interactions in Table 2.

Given the strong associations observed between heatwave exposure and asthma exacerbations, our study further explored the role of socioeconomic vulnerability in heat‐related health risks by using ADI and SVI as effect modifiers. The inclusion of these indices provided insights into neighborhood‐level disparities and complementary dimensions of socioeconomic vulnerability at the census tract and census block group levels. Interaction terms revealed significant results for SVI‐modified effects at the census tract level and ADI‐modified effects at the census block group level, suggesting a pronounced amplification of asthma exacerbation risks in pediatric populations when both heat wave exposure and high social vulnerability co‐occur (Table 3, Figure 6).

**Table 3:** Odds Ratios (ORs) and 95% Confidence Intervals (CIs) for Interaction Effects Between Heat Wave Indices (HIs) and Social Vulnerability (SVI) or Area Deprivation Indices (ADI), Stratified by Data Source, Spatial Resolution, and Age Group

Data source	Interaction term	Spatial resolution	Age group	Or and 95% CI
High Resolution	HI02*SVI	Census Tract	Pediatric	**1.18 (1.02, 1.38)**
High Resolution	HI02*SVI	Census Tract	Adult	**1.13 (1.05, 1.29)**
High Resolution	HI02*ADI	Census Block Group	Pediatric	1.03 (0.84, 1.27)
High Resolution	HI02*ADI	Census Block Group	Adult	1.05 (0.87, 1.27)
High Resolution	HI04*SVI	Census Tract	Pediatric	**1.26 (1.08, 1.48)**
High Resolution	HI04*SVI	Census Tract	Adult	**1.21 (1.07, 1.37)**
High Resolution	HI04*ADI	Census Block Group	Pediatric	**1.38 (1.07, 1.76)**
High Resolution	HI04*ADI	Census Block Group	Adult	**1.19 (1.01, 1.44)**
High Resolution	HI06*SVI	Census Tract	Pediatric	**1.20 (1.02, 1.42)**
High Resolution	HI06*SVI	Census Tract	Adult	**1.30 (1.07, 1.57)**
High Resolution	HI06*ADI	Census Block Group	Pediatric	**1.32 (1.05, 1.66)**
High Resolution	HI06*ADI	Census Block Group	Adult	1.12 (0.94, 1.33)
High Resolution	HI07*SVI	Census Tract	Pediatric	1.16 (0.90, 1.47)
High Resolution	HI07*SVI	Census Block Group	Pediatric	1.39 (0.96, 2.02)
NWS	HI01*SVI	Census Tract	Pediatric	**1.21 (1.04, 1.39)**
NWS	HI01*ADI	Census Block Group	Pediatric	**1.42 (1.14, 1.76)**
NWS	HI03*SVI	Census Tract	Pediatric	1.14 (0.96, 1.37)
NWS	HI03*SVI	Census Tract	Adult	1.13 (0.99, 1.27)
NWS	HI03*ADI	Census Block Group	Pediatric	1.32 (0.99, 1.73)
NWS	HI03*ADI	Census Block Group	Adult	1.20 (0.98, 1.44)
NWS	HI05*SVI	Census Tract	Pediatric	1.03 (0.87, 1.22)
NWS	HI05*ADI	Census Block Group	Pediatric	1.35 (1.01, 1.81)
NWS	HI07*ADI	Census Block Group	Pediatric	**1.66 (1.10, 2.48)**
NWS	HI08*SVI	Census Tract	Pediatric	1.14 (0.96, 1.37)
NWS	HI08*SVI	Census Tract	Adult	1.12 (0.99, 1.27)
NWS	HI08*ADI	Census Block Group	Pediatric	1.32 (0.97, 1.73)
NWS	HI08*ADI	Census Block Group	Adult	1.19 (0.99, 1.44)
NWS	HI09*SVI	Census Tract	Pediatric	1.04 (0.87, 1.23)
NWS	HI09*ADI	Census Block Group	Pediatric	1.35 (0.99, 1.81)

*Note*. Estimates with *p*‐values <0.05 are marked as bold. The table includes all combinations of heatwave definition, spatial resolution, and age group that yielded significant results in Table 2.

Our results using high‐resolution, 1‐m air temperature data show that pediatric asthma exacerbation risk was moderate but consistently elevated under nighttime based heat wave conditions in high‐SVI areas. For example, HI02*SVI (OR: 1.18; 95% CI: 1.02–1.38), HI04*SVI (OR: 1.26; 95% CI: 1.08–1.48), and HI06*SVI (OR: 1.20; 95% CI: 1.02–1.42), demonstrate significant interaction effects at the census tract level. At the census block group level, significant interaction effects were detected using ADI, including HI04*ADI (OR: 1.38; 95% CI: 1.07–1.76) and HI06*ADI (OR: 1.32; 95% CI: 1.05–1.66), indicating that pediatric patients living in areas with higher ADI scores faced elevated asthma risks during nighttime heat wave events. While the magnitude of these interactions was modest, the consistency across multiple minimum temperature derived HIs highlights the vulnerability of pediatric populations during sustained nighttime heat exposure. The elevated risks in high‐SVI and high‐ADI neighborhoods could arise through the distinct dimensions of vulnerability captured by these indices: SVI reflects social, demographic, and housing‐related disadvantage, while ADI reflects neighborhood‐level economic hardship. These forms of vulnerability are consistent with structural conditions observed in Baltimore's disadvantaged neighborhoods, including older housing stock, limited air‐conditioning access or use, and stronger nighttime heat island intensity, all of which can increase exposure to sustained nighttime heat. While ADI and SVI capture different factors relevant to exposure and vulnerability, the two indices are strongly correlated in Baltimore.

For adult patients, statistically significant interaction effects with SVI were also observed at the census tract level for HI02*SVI (OR: 1.13; 95% CI: 1.05–1.29), HI04*SVI (OR: 1.21; 95% CI: 1.07–1.37), and HI06*SVI (1.30; 95% CI: 1.07–1.57). At the census block group level, the interaction term HI04*ADI (OR: 1.19; 95% CI: 1.01–1.44) showed a 19% increased risk for asthma exacerbations in high‐ADI areas. Across both age groups, the significant interactions highlight the role of social vulnerability in modifying asthma risks during extreme heat events.

Using synoptic NWS air temperature data, evidence of effect modification by socioeconomic vulnerability was also observed, although fewer significant interactions were detected compared to high resolution air temperature data. For pediatric patients, a significant interaction between heatwave exposure and SVI was identified for HI01 (OR: 1.21; 95% CI: 1.04–1.39) at the census tract level. At the census block group level, significant interactions with ADI were found for HI01*ADI (OR: 1.42; 95% CI: 1.14–1.76) and HI07*ADI (OR: 1.66; 95% CI: 1.10–2.48), indicating elevated asthma risk during heatwaves among children living in areas of higher deprivation.

Comparing the results in Table 3 and Figure 6, we consider whether heatwaves defined using 1‐m air temperature data are more likely to resolve the mediating effects of socioeconomic vulnerability than are those than those defined using NWS data. For heatwaves defined at 1‐m resolution, interaction terms are positive and significant at 95% confidence for nine of the 14 models. For heatwaves defined using NWS data, only three of 14 models have significant interaction terms. This indicates that resolving the nighttime urban heat island in analysis of asthma vulnerability enhances our ability to detect the burden of heat in socially vulnerable neighborhoods of the urban core. While effect sizes were comparable between data sources, high‐resolution air temperature data demonstrated greater sensitivity in detecting socioeconomic effect modification.

Taken together, these findings suggest public health interventions should prioritize high‐SVI or high‐ADI areas, as these populations face compounded risks during heat waves. Given that nighttime heat exposure appears to be a key driver of asthma risk, strategies should focus on reducing in‐home heat during the night to mitigate these health impacts. While extending existing cooling center hours into the evening could provide additional cooling options, it is unlikely that individuals will remain in these locations overnight, limiting their effectiveness. Policies such as subsidizing residential air conditioning, expanding access to energy assistance programs, and improving housing infrastructure for heat resilience could be more effective in mitigating heat‐related health disparities. Additionally, implementing real‐time heat alerts that incorporate social vulnerability metrics can help direct resources to those most at risk, ensuring timely and effective responses to extreme heat events.

## 4 Conclusion

Regardless of how temperature is measured, whether using high‐resolution data or synoptic NWS measurements, extreme heat events consistently emerge as a risk factor for asthma in Baltimore City. Given this established relationship, the next critical step is to determine the most effective response strategies. However, the spatial resolution of temperature data influences our understanding of which extreme heat events pose the greatest risk. This highlights the need for careful consideration of spatial resolution and temperature metrics when assessing heat‐health research and assessing asthma risk.

This study provides compelling evidence that extreme heat exacerbates asthma in both pediatric and adult populations. By utilizing high‐resolution air temperature data and geocoded health records, we identified significant associations between asthma exacerbations and heat wave definitions that emphasize localized nighttime heat exposure, which is a particular risk factor in neighborhoods affected by the nocturnal UHI. These relationships were consistent across spatial resolutions, with stronger and more precise associations observed at the census block group level, demonstrating the value of granular data in capturing localized health impacts.

Our results also reveal important differences in how daytime and nighttime heatwaves relate to asthma risk, depending on the spatial resolution of the temperature data used. NWS synoptic station air temperature data captured significant associations between daytime maximum temperature‐based heat waves and asthma exacerbations, capturing broader regional heat impacts but missing the effects of nighttime heat exposure. This regional view is not as effective at detecting differences in the asthma‐heat burden related to within‐city differences in social vulnerability. Those mediating effects were more effectively captured in analyses that use 1‐m resolution air temperature estimates and that thus resolve the nocturnal urban heat island. These findings highlight the complementary nature of high and coarse resolution EOs. While NWS data are useful for identifying regional trends, high‐resolution data provide the granularity required to capture local heat impacts.

This study also found that Baltimore City's Code Red Extreme Heat alerts, which currently rely on a maximum daytime heat index, do not capture asthma risk and could benefit from incorporating nighttime heat wave definitions based on minimum temperatures. Elevated nighttime temperatures are associated with disrupted physiological recovery and disproportionately impact vulnerable populations such as children, the elderly, and those with pre‐existing conditions. Expanding the criteria for these alerts to include nighttime metrics would provide more comprehensive protection against heat‐related health risks and better align public health strategies with community needs.

Our findings highlight the disproportionate burden of extreme heat on socially and economically vulnerable populations. Significant interaction effects observed between heat wave definitions and social vulnerability indices revealed that patients in high‐SVI areas experienced asthma exacerbation risks increasing by as much as tenfold during extreme heat events. Similarly, higher ADI scores at the census block group were associated with increased asthma exacerbation risks, underscoring the importance of incorporating socioeconomic indicators in public health planning.

Several limitations to the present study should be noted. First, the high‐resolution data used in this study do not account for behavioral factors or individual‐level considerations, such as air conditioning use or other heat‐mitigation strategies. Second, while we demonstrated that the addition of relative humidity and air quality did not affect the estimate, unmeasured co‐exposures and confounders, such as pollen levels and indoor air quality could also impact asthma exacerbations. Further, prior research has shown that warmer conditions can amplify respiratory responses to pollutants such as ozone and fine particulate matter, suggesting that heat and air quality may jointly contribute to asthma risk in certain contexts (Pan et al., 2020). Although our study did not examine these co‐exposure pathways directly, they represent an important area for future work. Third, the health data were drawn from a single hospital system, which may introduce some limitations in representativeness across the broader city population. Lastly, the current study focuses exclusively on Baltimore, Maryland, and findings may not be directly generalizable to cities with different climatic or socioeconomic conditions.

Despite these limitations, this study emphasizes the importance of selecting appropriate heat wave definitions and utilizing high‐resolution EO and health data to address the impacts of extreme heat on vulnerable communities. These findings contribute to the growing body of evidence supporting targeted, equitable approaches to urban heat adaptation and health resilience, offering actionable insights to guide public health efforts.

## Conflict of Interest

The authors declare no conflicts of interest relevant to this study.

## Supporting information

Supporting Information S1

## Data Availability

Patient‐level emergency department data are not publicly available and cannot be shared by the authors because they contain protected health information, including hospital admission date, patient residential address, and patient residential Zip Code. The synoptic weather station air temperature data can be obtained from the NOAA's Maps and Geospatial Products (NOAA, 2024a). The high‐resolution air temperature data used in this analysis is publicly available (Corpuz et al., 2024). The air quality data can be obtained from the AirNow database (AirNow, 2024). The social vulnerability data can be obtained from the CDC Social Vulnerability Index database and University of Wisconsin's Neighborhood Atlas (Centers for Disease Control and Prevention, 2024; University of Wisconsin School of Medicine and Public Health, 2024).

## References

[gh270116-bib-0001] AirNow . (2024). Air quality index. Retrieved from https://www.airnow.gov/aqi/

[gh270116-bib-0002] Anderson, G. B. , & Bell, M. L. (2011). Heat waves in the United States: Mortality risk during heat waves and effect modification by heat wave characteristics in 43 U.S. Communities. Environmental Health Perspectives, 119(2), 210–218. 10.1289/ehp.1002313 21084239 PMC3040608

[gh270116-bib-0003] Baltimore City Health Department . (2017). 2017 neighborhood health profile for Baltimore City (pp. 1–33). Retrieved from https://health.baltimorecity.gov/sites/default/files/NHP%202017%20‐%2000%20Baltimore%20City%20(overall)%20(rev%206‐22‐17).pdf

[gh270116-bib-0004] Basu, R. , & Samet, J. M. (2002). Relation between elevated ambient temperature and mortality: A review of the epidemiologic evidence. Epidemiologic Reviews, 24(2), 190–202. 10.1093/epirev/mxf007 12762092

[gh270116-bib-0005] Bilheimer, L. T. , & Klein, R. J. (2010). Data and measurement issues in the analysis of health disparities: Disparities data and measurement. Health Services Research, 45(5p2), 1489–1507. 10.1111/j.1475-6773.2010.01143.x 21054368 PMC2965888

[gh270116-bib-0006] Buckley, J. P. , Samet, J. M. , & Richardson, D. B. (2014). Commentary: Does air pollution confound studies of temperature? Epidemiology, 25(2), 242–245. 10.1097/EDE.0000000000000051 24487206

[gh270116-bib-0007] CDC . (2023). Most recent national asthma data. Retrieved from https://www.cdc.gov/asthma/most_recent_national_asthma_data.htm

[gh270116-bib-0008] Centers for Disease Control and Prevention . (2024). CDC/ATSDR social vulnerability index database. Retrieved from https://www.atsdr.cdc.gov/placeandhealth/svi/data_documentation_download.html

[gh270116-bib-0009] Corpuz, B. , Zaitchik, B. , Waugh, D. , Scott, A. , & Logan, T. (2024). Shifting Islands: How weather conditions and urban form shape the spatiotemporal character of Baltimore's urban heat island. Urban Climate, 56, 102058. 10.1016/j.uclim.2024.102058

[gh270116-bib-0010] Cracknell, A. P. (2018). The development of remote sensing in the last 40 years. International Journal of Remote Sensing, 39(23), 8387–8427. 10.1080/01431161.2018.1550919

[gh270116-bib-0011] Deng, S. , Han, A. , Jin, S. , Wang, S. , Zheng, J. , Jalaludin, B. B. , et al. (2023). Effect of extreme temperatures on asthma hospital visits: Modification by event characteristics and healthy behaviors. Environmental Research, 226, 115679. 10.1016/j.envres.2023.115679 36913996

[gh270116-bib-0012] Dubovik, O. , Schuster, G. L. , Xu, F. , Hu, Y. , Bösch, H. , Landgraf, J. , & Li, Z. (2021). Grand challenges in satellite remote sensing. Frontiers in Remote Sensing, 2, 619818. 10.3389/frsen.2021.619818

[gh270116-bib-0013] Fang, J. , Song, J. , Wu, R. , Xie, Y. , Xu, X. , Zeng, Y. , et al. (2021). Association between ambient temperature and childhood respiratory hospital visits in Beijing, China: A time‐series study (2013–2017). Environmental Science and Pollution Research, 28(23), 29445–29454. 10.1007/s11356-021-12817-w 33555475

[gh270116-bib-0014] Flanagan, B. E. , Gregory, E. W. , Hallisey, E. J. , Heitgerd, J. L. , & Lewis, B. (2011). A social vulnerability index for disaster management. Journal of Homeland Security and Emergency Management, 8(1), 0000102202154773551792. 10.2202/1547-7355.1792

[gh270116-bib-0015] Frich, P. , Alexander, L. , Della‐Marta, P. , Gleason, B. , Haylock, M. , Klein Tank, A. , & Peterson, T. (2002). Observed coherent changes in climatic extremes during the second half of the twentieth century. Climate Research, 19, 193–212. 10.3354/cr019193

[gh270116-bib-0016] Gasparrini, A. , Guo, Y. , Hashizume, M. , Lavigne, E. , Zanobetti, A. , Schwartz, J. , et al. (2015). Mortality risk attributable to high and low ambient temperature: A multicountry observational study. The Lancet, 386(9991), 369–375. 10.1016/S0140-6736(14)62114-0 PMC4521077 26003380

[gh270116-bib-0017] Gong, P. , Wang, J. , Yu, L. , Zhao, Y. , Zhao, Y. , Liang, L. , et al. (2013). Finer resolution observation and monitoring of global land cover: First mapping results with Landsat TM and ETM+ data. International Journal of Remote Sensing, 34(7), 2607–2654. 10.1080/01431161.2012.748992

[gh270116-bib-0018] Han, A. , Deng, S. , Yu, J. , Zhang, Y. , Jalaludin, B. , & Huang, C. (2023). Asthma triggered by extreme temperatures: From epidemiological evidence to biological plausibility. Environmental Research, 216, 114489. 10.1016/j.envres.2022.114489 36208788

[gh270116-bib-0019] Hansen, A. , Bi, P. , Nitschke, M. , Ryan, P. , Pisaniello, D. , & Tucker, G. (2008). The effect of heat waves on mental health in a temperate Australian City. Environmental Health Perspectives, 116(10), 1369–1375. 10.1289/ehp.11339 18941580 PMC2569097

[gh270116-bib-0020] Hashimoto, M. , Fukuda, T. , Shimizu, T. , Watanabe, S. , Watanuki, S. , Eto, Y. , & Urashima, M. (2004). Influence of climate factors on emergency visits for childhood asthma attack. Pediatrics International, 46(1), 48–52. 10.1111/j.1442-200X.2004.01835.x 15043664

[gh270116-bib-0021] Hayes, D. , Collins, P. B. , Khosravi, M. , Lin, R.‐L. , & Lee, L.‐Y. (2012). Bronchoconstriction triggered by breathing hot humid air in patients with asthma: Role of cholinergic reflex. American Journal of Respiratory and Critical Care Medicine, 185(11), 1190–1196. 10.1164/rccm.201201-0088OC 22505744 PMC3373066

[gh270116-bib-0022] Heaviside, C. , Macintyre, H. , & Vardoulakis, S. (2017). The urban heat island: Implications for health in a changing environment. Current Environmental Health Reports, 4(3), 296–305. 10.1007/s40572-017-0150-3 28695487

[gh270116-bib-0023] Hondula, D. M. , Balling, R. C. , Vanos, J. K. , & Georgescu, M. (2015). Rising temperatures, human health, and the role of adaptation. Current Climate Change Reports, 1(3), 144–154. 10.1007/s40641-015-0016-4

[gh270116-bib-0024] Kent, S. T. , McClure, L. A. , Zaitchik, B. F. , Smith, T. T. , & Gohlke, J. M. (2014). Heat waves and health outcomes in Alabama (USA): The importance of heat wave definition. Environmental Health Perspectives, 122(2), 151–158. 10.1289/ehp.1307262 24273236 PMC3914868

[gh270116-bib-0025] Keshta, I. , & Odeh, A. (2021). Security and privacy of electronic health records: Concerns and challenges. Egyptian Informatics Journal, 22(2), 177–183. 10.1016/j.eij.2020.07.003

[gh270116-bib-0026] Kind, A. J. H. , & Buckingham, W. R. (2018). Making neighborhood‐disadvantage metrics accessible—The neighborhood atlas. New England Journal of Medicine, 378(26), 2456–2458. 10.1056/NEJMp1802313 29949490 PMC6051533

[gh270116-bib-0027] Konstantinoudis, G. , Minelli, C. , Lam, H. C. Y. , Fuertes, E. , Ballester, J. , Davies, B. , et al. (2023). Asthma hospitalisations and heat exposure in England: A case–crossover study during 2002–2019. Thorax, 78(9), 875–881. 10.1136/thorax-2022-219901 37068951 PMC10447396

[gh270116-bib-0028] Lu, Y. , Symons, J. M. , Geyh, A. S. , & Zeger, S. L. (2008). An approach to checking case‐crossover analyses based on equivalence with time‐series methods. Epidemiology, 19(2), 169–175. 10.1097/EDE.0b013e3181632c24 18223483

[gh270116-bib-0029] Lyon, B. (2009). Southern Africa summer drought and heat waves: Observations and coupled model behavior. Journal of Climate, 22(22), 6033–6046. 10.1175/2009JCLI3101.1

[gh270116-bib-0030] Maclure, M. (1991). The case‐crossover design: A method for studying transient effects on the risk of acute events. American Journal of Epidemiology, 133(2), 144–153. 10.1093/oxfordjournals.aje.a115853 1985444

[gh270116-bib-0031] Makrufardi, F. , Manullang, A. , Rusmawatiningtyas, D. , Chung, K. F. , Lin, S.‐C. , & Chuang, H.‐C. (2023). Extreme weather and asthma: A systematic review and meta‐analysis. European Respiratory Review, 32(168), 230019. 10.1183/16000617.0019-2023 37286218 PMC10245140

[gh270116-bib-0032] NOAA . (2024a). Maps and geospatial products [Dataset]. Retrieved from https://www.ncei.noaa.gov/maps‐and‐geospatial‐products

[gh270116-bib-0033] NOAA . (2024b). U.S. climate normals. Retrieved from https://www.ncei.noaa.gov/products/land‐based‐station/us‐climate‐normals

[gh270116-bib-0034] Pan, R. , Wang, X. , Yi, W. , Wei, Q. , Gao, J. , Xu, Z. , et al. (2020). Interactions between climate factors and air quality index for improved childhood asthma self‐management. Science of the Total Environment, 723, 137804. 10.1016/j.scitotenv.2020.137804 32213400

[gh270116-bib-0035] Pollack, C. E. , Roberts, L. C. , Peng, R. D. , Cimbolic, P. , Judy, D. , Balcer‐Whaley, S. , et al. (2023). Association of a housing mobility program with childhood asthma symptoms and exacerbations. JAMA, 329(19), 1671. 10.1001/jama.2023.6488 37191703 PMC10189571

[gh270116-bib-0036] PRISM Climate Group . (2024). PRISM gridded climate data. Oregon State University. Retrieved from https://prism.oregonstate.edu

[gh270116-bib-0037] Reid, C. E. , Snowden, J. M. , Kontgis, C. , & Tager, I. B. (2012). The role of ambient ozone in epidemiologic studies of heat‐related mortality. Environmental Health Perspectives, 120(12), 1627–1630. 10.1289/ehp.1205251 22899622 PMC3548272

[gh270116-bib-0038] Rey, G. , Jougla, E. , Fouillet, A. , Pavillon, G. , Bessemoulin, P. , Frayssinet, P. , et al. (2007). The impact of major heat waves on all‐cause and cause‐specific mortality in France from 1971 to 2003. International Archives of Occupational and Environmental Health, 80(7), 615–626. 10.1007/s00420-007-0173-4 17468879 PMC2291483

[gh270116-bib-0039] Robinson, P. J. (2001). On the definition of a heat wave. Journal of Applied Meteorology, 40(4), 762–775. 10.1175/1520-0450(2001)040<0762:OTDOAH>2.0.CO;2

[gh270116-bib-0040] Rothfusz, L. P. (1990). The heat index “Equation” (or, more than you ever wanted to know about heat index).

[gh270116-bib-0041] Russo, S. , Sillmann, J. , & Sterl, A. (2017). Humid heat waves at different warming levels. Scientific Reports, 7(1), 7477. 10.1038/s41598-017-07536-7 28785096 PMC5547064

[gh270116-bib-0042] Schinasi, L. H. , Kenyon, C. C. , Hubbard, R. A. , Zhao, Y. , Maltenfort, M. , Melly, S. J. , et al. (2022). Associations between high ambient temperatures and asthma exacerbation among children in Philadelphia, PA: A time series analysis. Occupational and Environmental Medicine, 79(5), 326–332. 10.1136/oemed-2021-107823 35246484

[gh270116-bib-0043] Shi, R. , Hobbs, B. F. , Zaitchik, B. F. , Waugh, D. W. , Scott, A. A. , & Zhang, Y. (2021). Monitoring intra‐urban temperature with dense sensor networks: Fixed or mobile? An empirical study in Baltimore, MD. Urban Climate, 39, 100979. 10.1016/j.uclim.2021.100979

[gh270116-bib-0044] Smith, T. T. , Zaitchik, B. F. , & Gohlke, J. M. (2013). Heat waves in the United States: Definitions, patterns and trends. Climatic Change, 118(3–4), 811–825. 10.1007/s10584-012-0659-2 23869115 PMC3711804

[gh270116-bib-0045] Soneja, S. , Jiang, C. , Fisher, J. , Upperman, C. R. , Mitchell, C. , & Sapkota, A. (2016). Exposure to extreme heat and precipitation events associated with increased risk of hospitalization for asthma in Maryland, U.S.A. Environmental Health, 15(1), 57. 10.1186/s12940-016-0142-z 27117324 PMC4847234

[gh270116-bib-0046] Steadman, R. G. (1979). The assessment of sultriness. Part I: A temperature‐humidity index based on human physiology and clothing science. Journal of Applied Meteorology, 18(7), 861–873. 10.1175/1520-0450(1979)018<0861:TAOSPI>2.0.CO;2

[gh270116-bib-0047] University of Wisconsin School of Medicine and Public Health . (2024). Area deprivation index. Retrieved from https://www.neighborhoodatlas.medicine.wisc.edu

[gh270116-bib-0048] U.S. Census Bureau . (2020). Maryland: 2020 Census. Retrieved from https://www.census.gov/quickfacts/fact/table/US,baltimorecitymaryland/PST045221

[gh270116-bib-0049] Yu, J. , Zhu, A. , Liu, M. , Dong, J. , Tian, T. , Liu, T. , et al. (2024). The correlation between daily temperature, diurnal temperature range, and asthma hospital admissions in Lanzhou city, 2013–2020. BMC Public Health, 24(1), 2454. 10.1186/s12889-024-19737-7 39251927 PMC11386359

