# Supplementary material for: Impact of Extreme Heat on Emergency Department Admissions for Childhood and Adult Asthma: An Evaluation of Earth Observations and Heat Wave Definitions
Source: Geohealth. 2026 May 6;10(5):e2025GH001501. doi: 10.1029/2025GH001501 (PMC13147955; doi:10.1029/2025GH001501)
Supplement: Supplementary file 1 — Supporting Information S1 [file GH2-10-e2025GH001501-s001.docx]

*GeoHealth*

Supporting Information for

**Impact of Extreme Heat on Emergency Department Admissions for Childhood and Adult Asthma: An Evaluation of Earth Observations and Heat Wave Definitions**

B. Corpuz^1^, E. Scott^2^, B. F. Zaitchik^1^, S. Zeger^2^, D. Waugh^1^, A. Balasubramanian^3^, J. Madrigano^4^, K. Koehler^4^, R. Koehl^3^, and M. McCormack^3^

^1^ Department of Earth and Planetary Sciences, Johns Hopkins University, Baltimore, United States

^2^ Department of Biostatistics, Johns Hopkins University Bloomberg School of Public Health, Baltimore, United States

^3^ Division of Pulmonary and Critical Care Medicine, Johns Hopkins University School of Medicine, Baltimore, United States

^4^ Department of Environmental Health and Engineering, Johns Hopkins University Bloomberg School of Public Health, Baltimore, United States

**Contents of this file**

Figures S1 to S4

Table S1


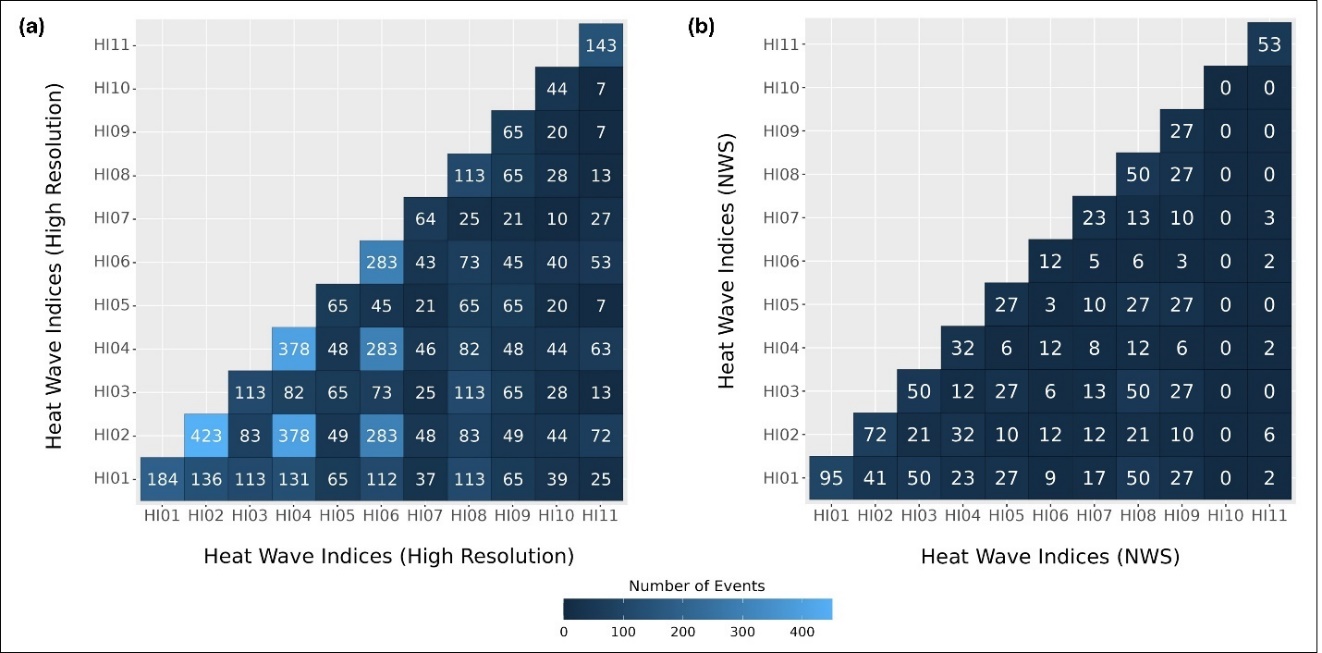


Figure S1. Co-occurrence matrices of heat wave events based on the Heat Waves Indices (HIs) used in this study, calculated using (a) high-resolution, 1-meter air temperature data, and (b) synoptic NWS air temperature data. Each cell represents the number of heat wave events that co-occurred on the same dates during summer months, June, July, and August, from 2016-2022 in Baltimore.


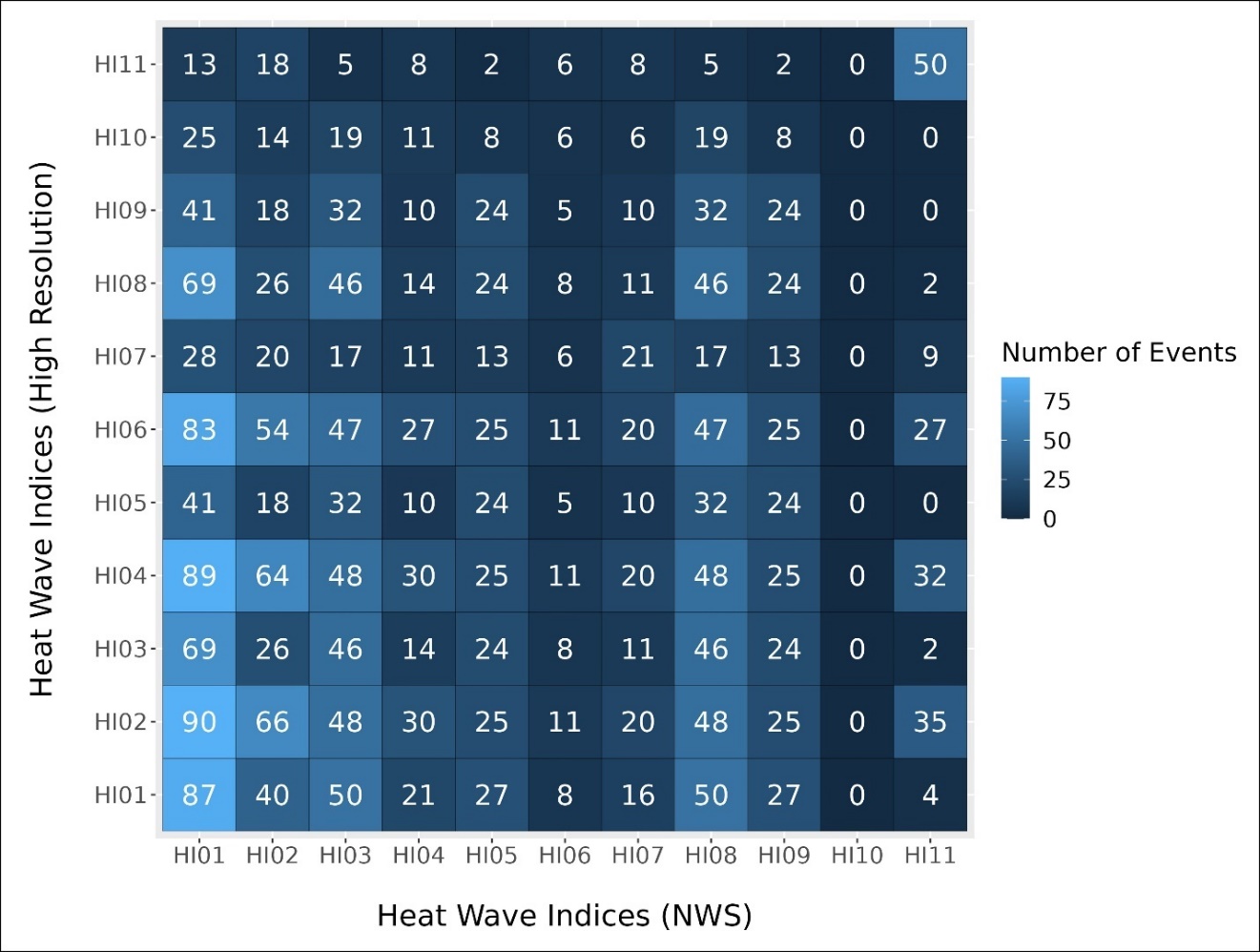


Figure S2. Cross-source co-occurrence matrix of heat wave events based on the Heat Waves Indices (HIs) used in this study, comparing events identified using high-resolution, 1-meter air temperature data and synoptic NWS air temperature data. Each cell represents the number of heat wave events that were identified by both the high-resolution and NWS-based HIs on the same dates during summer months, June, July, and August, from 2016-2022 in Baltimore.


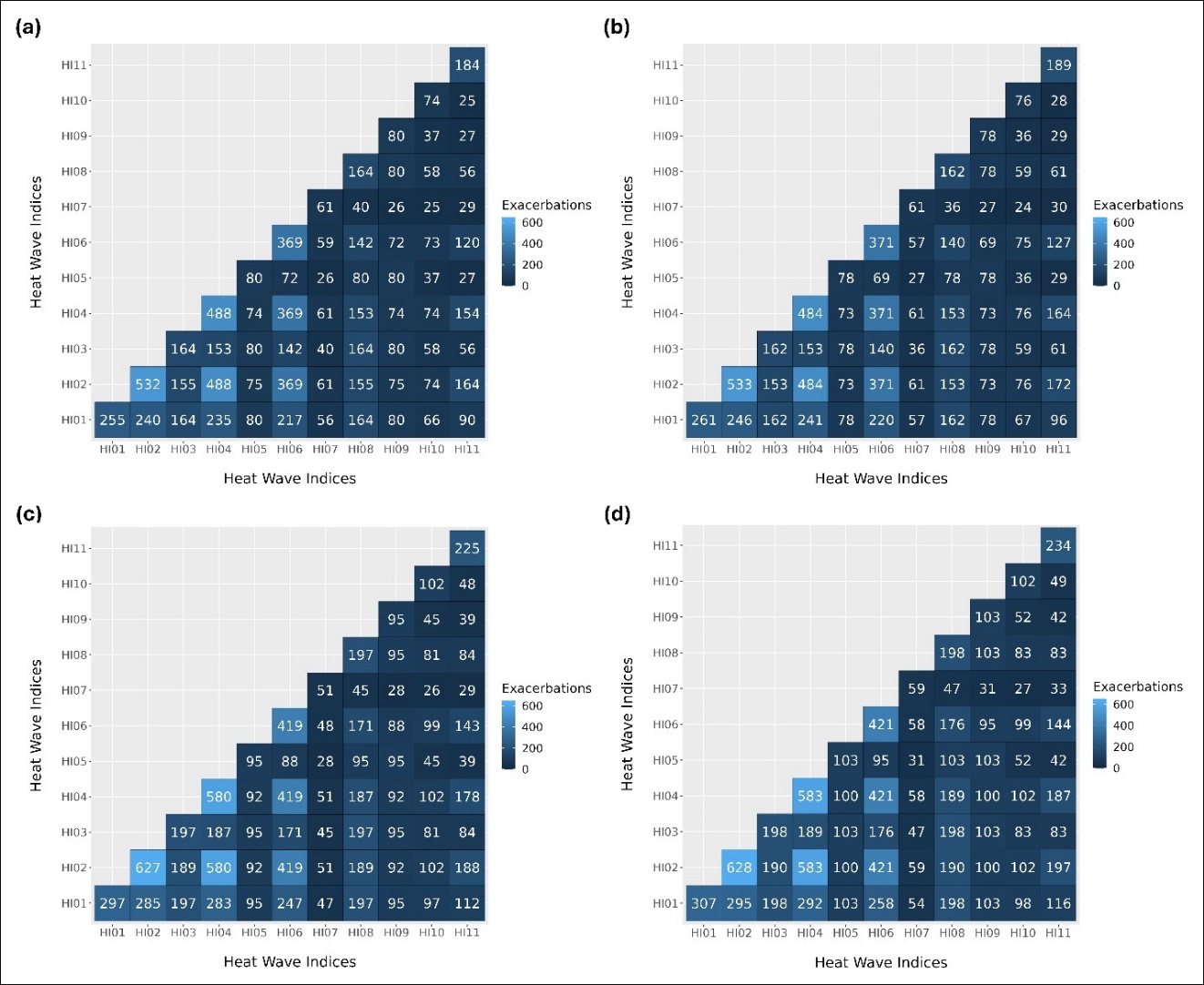


**Figure S3.** Co-occurrence matrices of asthma exacerbation count associated with Heat Wave Indices (HIs) defined using high-resolution, 1-meter air temperature data. Each cell represents the number of asthma exacerbations that occurred during overlapping heat wave events defined by each HI pair. Panels show results stratified by age group and spatial resolution: pediatric (a, b) and adult (c, d) patients, at the census tract (a, c) and census block group (b, d) levels.


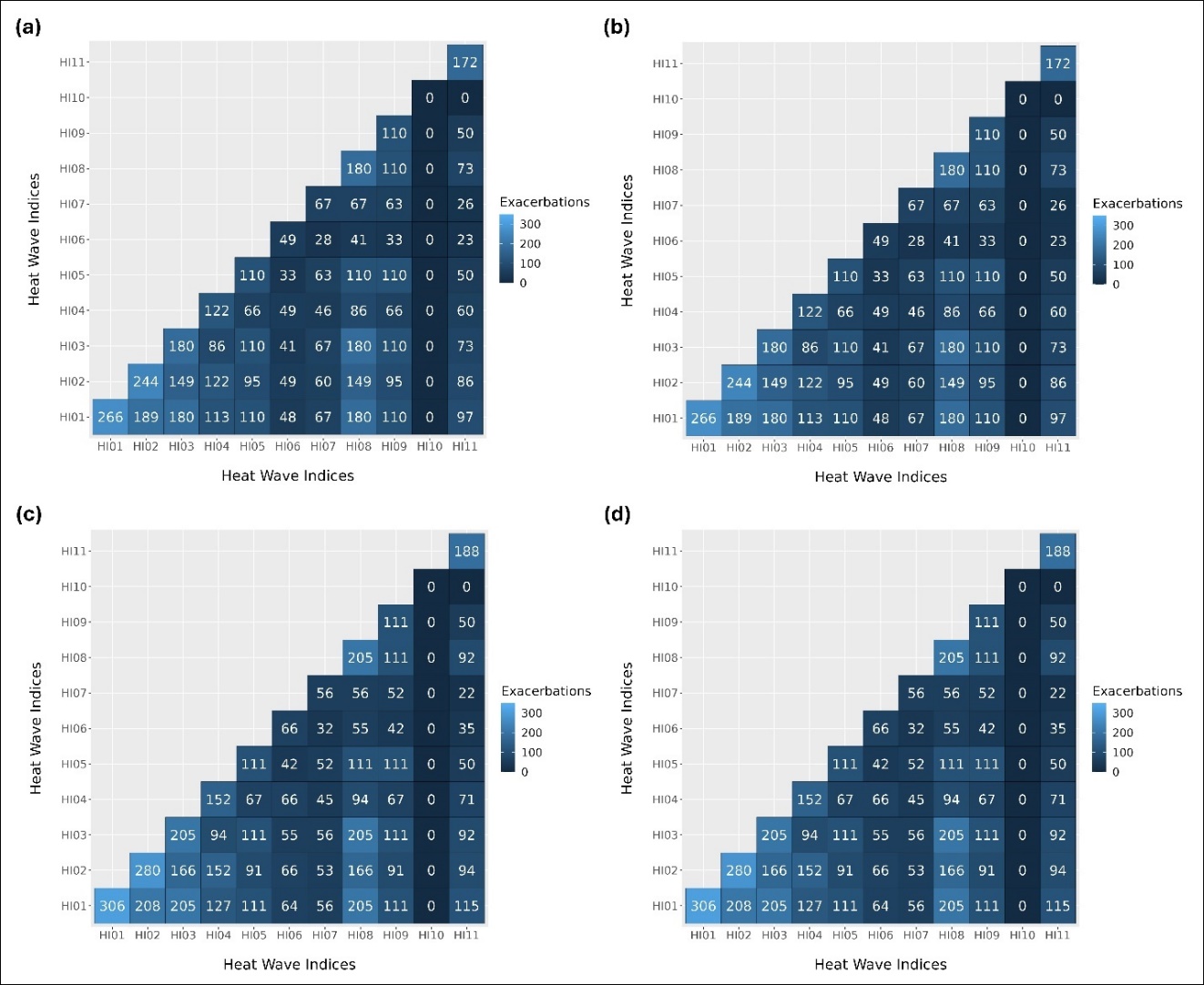


**Figure S4.** Co-occurrence matrices of asthma exacerbation count associated with Heat Wave Indices (HIs) defined using synoptic NWS air temperature data. Each cell represents the number of asthma exacerbations that occurred during overlapping heat wave events defined by each HI pair. Panels show results stratified by age group and spatial resolution: pediatric (a, b) and adult (c, d) patients, at the census tract (a, c) and census block group (b, d) levels.

**Table S1.** Odds ratios (ORs) and 95% confidence intervals (CIs) for asthma exacerbations, adjusted for AQI and relative humidity, by heat index definitions (HIs) and resolution for pediatric and adult patients in Baltimore, from 2016 to 2022. High resolution corresponds to HI definitions derived from 1-m air temperature data, while NWS refers to National Weather Service (NWS) air temperature data from the BWI airport station. Estimates with p-values < 0.05 are marked as bold. A dash indicates insufficient data available.

*Note:* Rows corresponding to nighttime temperature-based heat indices (HI02, HI04, HI06) are lightly shaded to aid visual interpretation.

| **Heat Wave Indices (HI)** | **Spatial Resolution** | **Age Group** | **OR & 95% CI**  **(High Resolution)** | **OR & 95% CI**  **(NWS)** |
| --- | --- | --- | --- | --- |
| HI01 | Census Tract | Pediatric | 0.94 (0.77, 1.15) | **1.32 (1.07, 1.64)** |
| HI01 | Census Tract | Adult | 0.88 (0.73, 1.06) | 1.06 (0.87, 1.29) |
| HI01 | Census Block Group | Pediatric | 0.98 (0.80, 1.20) | **1.32 (1.07, 1.64)** |
| HI01 | Census Block Group | Adult | **0.82 (0.68, 0.99)** | 1.06 (0.87, 1.29) |
| HI02 | Census Tract | Pediatric | **1.31 (1.03, 1.67)** | 1.09 (0.87, 1.37) |
| HI02 | Census Tract | Adult | **1.28 (1.02, 1.59)** | 1.22 (0.99, 1.52) |
| HI02 | Census Block Group | Pediatric | **1.35 (1.06, 1.72)** | 1.09 (0.87, 1.37) |
| HI02 | Census Block Group | Adult | **1.26 (1.01, 1.57)** | 1.22 (0.99, 1.52) |
| HI03 | Census Tract | Pediatric | 1.03 (0.82, 1.29) | **1.56 (1.22, 1.99)** |
| HI03 | Census Tract | Adult | 0.91 (0.74, 1.12) | **1.28 (1.02, 1.60)** |
| HI03 | Census Block Group | Pediatric | 0.99 (0.79, 1.24) | **1.56 (1.22, 1.99)** |
| HI03 | Census Block Group | Adult | 0.88 (0.72, 1.09) | **1.28 (1.02, 1.60)** |
| HI04 | Census Tract | Pediatric | **1.38 (1.09, 1.75)** | 0.97 (0.75, 1.24) |
| HI04 | Census Tract | Adult | **1.36 (1.10, 1.69)** | 0.98 (0.78, 1.23) |
| HI04 | Census Block Group | Pediatric | **1.28 (1.01, 1.62)** | 0.97 (0.75, 1.24) |
| HI04 | Census Block Group | Adult | **1.40 (1.13, 1.74)** | 0.98 (0.78, 1.23) |
| HI05 | Census Tract | Pediatric | 1.07 (0.80, 1.44) | **1.50 (1.15, 1.95)** |
| HI05 | Census Tract | Adult | 0.89 (0.68, 1.16) | 1.23 (0.95, 1.59) |
| HI05 | Census Block Group | Pediatric | 0.97 (0.72, 1.30) | **1.50 (1.15, 1.95)** |
| HI05 | Census Block Group | Adult | 0.98 (0.76, 1.28) | 1.23 (0.95, 1.59) |
| HI06 | Census Tract | Pediatric | **1.34 (1.07, 1.68)** | 1.00 (0.70, 1.43) |
| HI06 | Census Tract | Adult | **1.30 (1.05, 1.60)** | 1.05 (0.77, 1.43) |
| HI06 | Census Block Group | Pediatric | **1.30 (1.04, 1.63)** | 1.00 (0.70, 1.43) |
| HI06 | Census Block Group | Adult | **1.31 (1.06, 1.62)** | 1.05 (0.77, 1.43) |
| HI07 | Census Tract | Pediatric | **1.62 (1.15, 2.28)** | **1.75 (1.25, 2.43)** |
| HI07 | Census Tract | Adult | 1.05 (0.74, 1.49) | 1.07 (0.77, 1.48) |
| HI07 | Census Block Group | Pediatric | **1.47 (1.05, 2.06)** | **1.75 (1.25, 2.43)** |
| HI07 | Census Block Group | Adult | 1.14 (0.82, 1.59) | 1.07 (0.77, 1.48) |
| HI08 | Census Tract | Pediatric | 1.03 (0.82, 1.29) | **1.56 (1.22, 1.99)** |
| HI08 | Census Tract | Adult | 0.91 (0.74, 1.12) | **1.28 (1.02, 1.60)** |
| HI08 | Census Block Group | Pediatric | 0.99 (0.79, 1.24) | **1.56 (1.22, 1.99)** |
| HI08 | Census Block Group | Adult | 0.88 (0.72, 1.09) | **1.28 (1.02, 1.60)** |
| HI09 | Census Tract | Pediatric | 1.07 (0.80, 1.44) | **1.50 (1.15, 1.95)** |
| HI09 | Census Tract | Adult | 0.89 (0.68, 1.16) | 1.23 (0.95, 1.59) |
| HI09 | Census Block Group | Pediatric | 0.97 (0.72, 1.30) | **1.50 (1.15, 1.95)** |
| HI09 | Census Block Group | Adult | 0.98 (0.76, 1.28) | 1.23 (0.95, 1.59) |
| HI10 | Census Tract | Pediatric | 1.23 (0.89, 1.70) | - |
| HI10 | Census Tract | Adult | 0.93 (0.70, 1.24) | - |
| HI10 | Census Block Group | Pediatric | 1.25 (0.90, 1.72) | - |
| HI10 | Census Block Group | Adult | 0.92 (0.69, 1.21) | - |
| HI11 | Census Tract | Pediatric | 0.95 (0.76, 1.18) | 1.31 (0.98, 1.65) |
| HI11 | Census Tract | Adult | 0.95 (0.78, 1.17) | 1.00 (0.81, 1.23) |
| HI11 | Census Block Group | Pediatric | 0.93 (0.75, 1.15) | 1.31 (0.98, 1.65) |
| HI11 | Census Block Group | Adult | 0.96 (0.78, 1.17) | 1.00 (0.81, 1.23) |
